# An ontology for representing hematologic malignancies: the cancer cell ontology

**DOI:** 10.1186/s12859-019-2722-8

**Published:** 2019-04-25

**Authors:** Lucas M. Serra, William D. Duncan, Alexander D. Diehl

**Affiliations:** Department of Biomedical Informatics, Jacobs School of Medicine and Biomedical Sciences, University at Buffalo, Buffalo, NY USA

**Keywords:** Blood cancer, Leukemia, Cell ontology, Cancer cell ontology, CCL

## Abstract

**Background:**

Within the cancer domain, ontologies play an important role in the integration and annotation of data in order to support numerous biomedical tools and applications. This work seeks to leverage existing standards in immunophenotyping cell types found in hematologic malignancies to provide an ontological representation of them to aid in data annotation and analysis for patient data.

**Results:**

We have developed the Cancer Cell Ontology according to OBO Foundry principles as an extension of the Cell Ontology. We define classes in Cancer Cell Ontology by using a genus-differentia approach using logical axioms capturing the expression of cellular surface markers in order to represent types of hematologic malignancies. By adopting conventions used in the Cell Ontology, we have created human and computer-readable definitions for 300 classes of blood cancers, based on the EGIL classification system for leukemias, and relying upon additional classification approaches for multiple myelomas and other hematologic malignancies.

**Conclusion:**

We have demonstrated a proof of concept for leveraging the built-in logical axioms of the ontology in order to classify patient surface marker data into appropriate diagnostic categories. We plan to integrate our ontology into existing tools for flow cytometry data analysis to facilitate the automated diagnosis of hematologic malignancies.

## Background

### Preface

We live in an age of ever-increasing troves of data comprised of genomic, imaging and clinical information. Within the field of medicine, oncology leads in this regard by using large, varied datasets to refine therapies and stratify patient populations into meaningful subgroups. The explosion of data heralds new challenges for researchers and technologists that struggle to keep track of these mountains of information while maintaining interoperability between systems. [1] One of the methods to manage, sort, and analyze this data comes in the form of ontologies, which are representational artifacts comprised of universals and the relations between them that designate entities in reality. [2] Briefly, ontologies at their core are semantic terminologies that exist as two types: reference ontologies, which embody established knowledge via rich, precise meanings for terms in a domain, and application ontologies, which are designed for a specific purpose and weave together sets of related classes from reference ontologies in order to represent the entities of complex domains. [3, 4]

### The cell ontology

A description of past ontologies that represent cells is warranted as the work presented here directly builds upon these artifacts. The Cell Ontology (CL) was originally developed in 2005 with the goal of representing a variety of cell types from the prokaryotic, fungal, animal and plant worlds. [5] As interest and support has shifted over the years, the scope of the CL has shifted to focus primarily on vertebrate cell types with special attention to hematopoietic cell types. [6, 7] The CL links to other ontologies within the Open Biological and Biomedical Ontology (OBO) foundry via relations from the Relations Ontology. [8, 9] These relations often take the form of *has_plasma_membrane_part* to connect cell types to appropriate surface markers found in the Protein Ontology. Similarly, the relations, *has_high_plasma_membrane_amount* and *has_low_plasma_membrane_amount* are used within computable definitions to denote surface protein expression that is above or below the mean of a population of cells and were originally described in Masci et al., and generally relate to relative expression values determined by flow cytometry. [10] Lastly, negative criteria are also implemented in definitions using *lacks_part* and *lacks_plasma_membrane_part* relations.

### Cancer ontologies

Within the cancer domain, ontologies are an important component of numerous biomedical tools and applications. Without question, the most successful ontology in cancer research is the Gene Ontology (GO) owing to its widespread use. A PubMed search using the search terms “cancer”, “oncology” and “gene ontology” reveals hundreds of articles published within the last five years. Even after excluding the GO, it is apparent a number of diverse ontologies have seen varied application in cancer research. Longstanding ontologies have been used to annotate and integrate oncologic data. The Foundational Model of Anatomy has been used to annotate biomarkers for brain tumors while the Disease Ontology (DO) has been used to integrate several databases into a cohesive set. [11, 12] Newer ontologies have cropped up in recent years to represent the many facets of cancer care including ontologies representing staging systems (TMN ontology), cancer treatments, brachytherapy (ENT COBRA ontology), and after-care treatment plans that enhance patient engagement (Profile Ontology for Adolescent and Young Adult Cancer Survivors). [13–16] Additionally, ontologies support text mining applications, clinical decision support systems, the analyzing of adverse events, and the targeting of cancer drugs. [17–20] The National Cancer Institute (NCI) Thesaurus is one of the largest and most widely used resources within the field of cancer. Although technically a terminology with ontology-like features, the NCI Thesaurus covers 110,000 terms in 36,000 concepts in the cancer research domain and arose from a need to integrate varied data systems through a unified coding system. [21, 22]

### Diagnosing blood cancers

The diagnosis of hematologic malignancies like acute lymphoblastic leukemia (ALL) and acute myeloid leukemia (AML) involves a myriad of tests and clinical examination ranging from a simple history and physical to advanced genetic testing. In terms of pathological assessment, patient blood samples or bone marrow aspirates are stained and examined for the morphology of cancer cells. Karyotyping provides information on chromosomal translocations. Recently, genetic analysis either through microarrays or sequencing provides detailed insight into the molecular characterizations of cell populations. [23] Flow cytometry is another mainstay of blood cancer diagnosis whereby a laser examines the emission properties of cells labeled with fluorochrome-conjugated antibodies in a suspension. These antibodies are specific for cell markers of interest and are commercially available as a product that contains an attached fluorochrome. The flow cytometer is capable of assessing the expression of markers, which are typically surface proteins, on millions of cells in real-time. [24] The output of this process is referred to as the immunophenotype of a cell. Each antibody-fluorochrome conjugate can emit a different range of light wavelengths allowing for simultaneous assessment of multiple markers. Laboratories apply antibody panels to patient specimens to typically examine four to eight different markers at a time although the use of larger panels is increasingly possible as technology improves. The definition of positivity for markers has changed over time as technical sensitivity has improved. In the past, a simple cutoff of 20% of the population bearing a marker was labeled as positive. Increasingly, this method has been superseded by comparing the fluorescence shift and distribution pattern of cancer cell populations to appropriate controls. [25]

In the current work, we have created an ontology, the Cancer Cell Ontology (CCL), that represents cancer cell types in the domain of hematologic malignancies, namely acute lymphoblastic leukemia, acute myeloid leukemia and multiple myeloma, using immunophenotypes as differentia.

## Methods

The CCL was created with the latest version of Protégé (5.2.0) developed by the Stanford Center for Biomedical Informatics Research. [26] Our ontology imports the entirety of the CL, which indirectly imports modules from the Protein Ontology (PRO), the Chemical Entities of Biological Interest ontology (CHEBI), the Phenotypic Quality Ontology (PATO), the Cell Line Ontology (CLO), the Relation Ontology (RO), the National Center for Biotechnology Information taxonomy (NCIT), the Uber Anatomy Ontology (UBERON) and the GO. The CCL also directly imports small OWL modules from PRO and CHEBI. [9, 27–33] The total size of the CCL as viewed in Protégé is 6900 classes. 6600 of these classes have been imported from the CL and 300 new terms have been added. Roughly three dozen terms were reused from the CL and primarily consist of PRO terms. The ELK 0.4.3 reasoner was used for inferential reasoning. [34] All terms added by the CCL have been manually reviewed for errors of inconsistency. Additionally, Protégé’s built-in debugging tool found no errors. A GitHub page with the latest version of the ontology is available at: https://github.com/LucasSerra1/CCL.git for viewing.

## Results

### Classification systems

The CCL was constructed according to published guidelines of best practices in ontology development and adheres to the principles put forth by the OBO foundry such as openness, a common format, textual definitions, well-defined relations, etc. [2, 8] A genus-differentia approach was taken to construct the new classes by using surface marker expression as the main axis. There exist many schemas for classifying leukemia with systems such as WHO classification, the French–American–British classification system, St. Jude’s system and the European Group for Immunophenotyping Leukemia (EGIL) system. [35–37] The EGIL system was selected as the backbone hierarchy for the ontology due to a few compelling factors. EGIL does not represent simply a single institution’s idea of leukemia. The EGIL system is the continuously developing result of years of discussion and consensus from what is now known as the Euroflow Consortium, a group composed of more than forty researchers spanning eight nations that began in 2006. [38] As a consequence, this system has seen widespread use in laboratories and initial proposals have seen exorbitant numbers of citations. [39] Additionally, this leukemia classification system relies exclusively on the immunophenotypes of leukemic cells, which is precisely the information we wish to capture in our ontology. Table 1 is a diagram of the overarching structure of the EGIL system.

**Table 1:** EGIL classification scheme

EGIL classification system	
Immunologic subgroup	Immunophenotypic profile
B-lineage ALL	CD19+ and/or CD79a + and/or CD22+
B-I (pro-B)	No B-cell differentiation antigens
B-II (common B)	CD10+
B-III (pre-B)	cyIgμ+
B-IV (mature B)	cylg or sIg λ + or κ+
T-lineage ALL	Cytoplasmic/surface CD3+
T-I (pro-T)	CD7+
T-II (pre-T)	CD2+ and/or CD5+ and/or CDS+
T-III (cortical T)	CD1a+
T-IV (mature T)	Surface CD3+, CD la-
α/β (group a)	TCR α/β+
γ/δ (group b)	TCR γ/δ+
Early myeloid (AML-MO)	MPO ± but enzymatic MPO−/CD13+/CD33+/ CD65+/and-or CD 117+
Myelo/monocytic lineage	MPO+/CD13+/CD33+/CD65+/and-or CD117+
Megakaryocytic lineage	CD41+ and/or CD61+ (surface or cytoplasmic)
Erythroid lineage	Early/immature: unclassified by markers Late/mature: GPA+
Undifferentiated	Often CD34+/HLA-DR+/CD38+/CD7+

Unfortunately, there exists no such unified classification system for other blood cancers like multiple myeloma. Instead, we pooled several antibody panels geared towards the diagnosis of multiple myeloma that had been examined in recent literature. The consensus of four separate studies, which included review articles, was used to create the classes of multiple myeloma within our ontology. [40–43]

### Definitions, relations, and structure

**Fig. 1:**
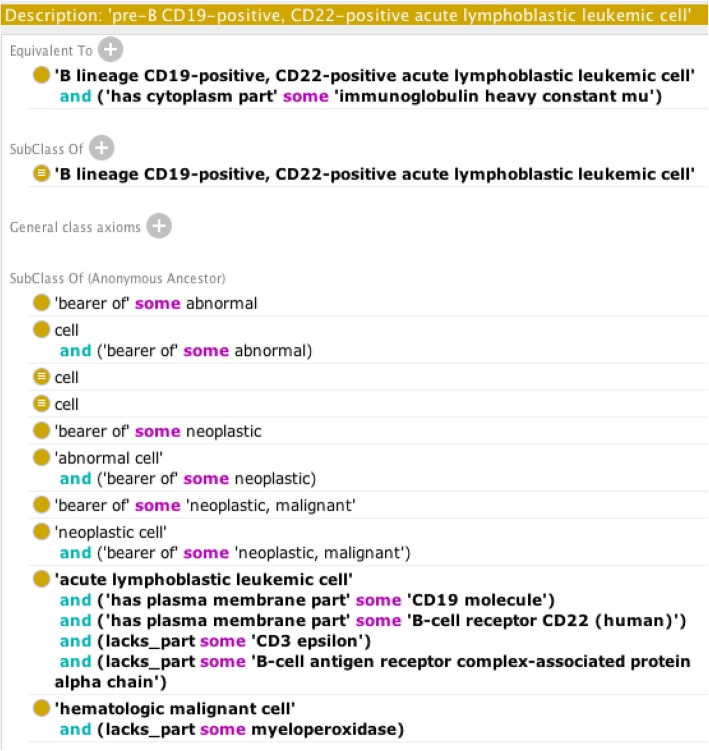
Machine-readable definition from the CCL

The CCL exists as an extension of the CL. The root term of the CCL ‘hematologic malignant cell’ resides under the CL parent term ‘malignant cell’, which exists under the terms ‘neoplastic cell’ and ‘abnormal cell’. Our ontology contains human and machine-readable definitions for every added class. Many of these definitions follow a similar format to definitions found in the CL by linking necessary surface markers to the corresponding elements in the Protein Ontology. The textual definitions of the CCL upper level terms are defined according to the cell lineage the aberrant cell is derived from. For instance, ‘acute lymphoblastic leukemic cell’ is defined as a “hematologic malignant cell whose precursor is of lymphoid lineage”. Lower level child terms are instead textually defined according to marker expression as seen in the definition of ‘pre-B CD19-positive, CD22-positive acute lymphoblastic leukemic cell’, which states that this entity is a “B lineage CD19-positive, CD22-positive acute lymphoblastic leukemic cell that is cytoplasmic Ig mu positive”. The axiomatic definitions follow the same structure and use relations such as *has_plasma_membrane_part*, *has_cytoplasm_part* and *lacks_part* to denote marker positivity status (Fig. 1). The CCL also includes two new relations, *has_cytoplasm_part* and *lacks_cytoplasm_part*. These relations are absent from the imported ontologies and were needed to represent the handful of cases that use cytoplasmic markers to distinguish classes. The definitions of these relations mirror their plasma membrane counterparts from the CL as *has_cytoplasm_part* is defined as holding “between a cell **c** and a protein complex or protein **p** if and only if that cell has as part a cytoplasm, and that cytoplasm has **p** as part,” which means that if a protein is found in the cytoplasm of a cell then that cell has the protein as a cytoplasmic part.

The CCL contains over 300 classes representing cancer cell types found in acute lymphoblastic leukemia, acute myeloid leukemia and multiple myeloma, which are based upon widely used classification systems and pooled studies. Each class is differentiated based upon surface marker expression and has human and machine-readable definitions that are composed of necessary and sufficient conditions. We have also created test instances of patient data with a series of positive or negative marker expression. After running the reasoner, the test data was sorted into matching CCL classes, which demonstrates the utility of our ontology towards automatically sorting patient cell data into relevant diagnostic groups.

## Discussion

The CCL is a natural evolution of the work started on hematopoietic cells in the CL and is the first ontology to represent the cell types of hematologic malignancies. This ontology contributes to the process of cancer research in a number of ways. This is new work that can complement work that already exists in the NCIT and DO. Our ontology enables sophisticated queries on patient data and allows researchers to efficiently examine surface marker expression. This approach could allow for easier stratification of novel subgroups of patients. The CCL also enables easier integration of disparate data sources by providing a structured semantic representation and explicit, well-crafted definitions that are human and computer readable. With respect to the antibody panels themselves, our ontology lends itself to the creation of a separate antibody panel ontology that could allow researchers to determine whether there exists agreement across laboratories in regards to antibody panel composition and the definition of cancer cell types based on reactivity to the panels. We realize our focus on immunophenotypes paints an incomplete picture of blood cancer cells and omits important diagnostic and prognostic information regarding these malignancies. It is our plan that future development of the CCL includes integration of cytogenetic and morphological logical definitions and allows for utilization of patient-specific information. This would, in turn, allow for a more holistic description of blood cancer cells and enable more accurate classifications of malignancies to further subtype patients.

The sheer number and diversity of surface markers also presents an issue to future development of the CCL. As each individual class is defined as some combination of surface marker status, adding new surface markers increases the work of adding new classes exponentially. Luckily, current panels at most use eight antibody-conjugates, but with additional markers we see a combinatorial explosion in terms of class definitions. In the same vein, every cancer patient is unique and each disease phenotype is unique. However, there appear to be common patterns of marker expression for the various cancer types, and deep phenotyping may reveal important subgroups that relate to speed of disease progression and responsiveness to treatments.

Perhaps most importantly, we have shown a proof of concept for leveraging the built-in logical axioms of the ontology in order to classify patient surface marker data into appropriate diagnostic categories. The current work will eventually be part of a larger framework involving a combination of FLOCK clustering analysis of the raw flow cytometry data in combination with the flowCL tool, which will be modified to match cell types to corresponding entities in the CL and CCL. [44, 45] By incorporation CCL into this software system we hope to facilitate the automated diagnosis of blood cancers. Future iterations of the CCL will incorporate additional classification systems and represent a broader range of blood cancers. We also plan to relate each cancer cell type to its immediate normal precursor in the style of the CL.

## Conclusion

The CCL is the first ontology to represent hematologic malignancies solely via their immunophenotypes and succeeds as a first step towards increased automation in the diagnosis of blood cancers. We plan to integrate our ontology into existing tools for flow cytometry data analysis to facilitate the automated diagnosis of hematologic malignancies.
